# The influence of vitamin D on handgrip strength in elderly trauma patients

**DOI:** 10.1186/s40001-023-01123-5

**Published:** 2023-05-13

**Authors:** Tamara Ostermeier, Leon Faust, Adrian Cavalcanti-Kußmaul, Christian Kammerlander, Matthias Knobe, Wolfgang Böcker, Maximilian M. Saller, Carl Neuerburg, Alexander M. Keppler

**Affiliations:** 1 Department of Orthopaedic and Trauma Surgery, Musculoskeletal University Center Munich (MUM), University Hospital LMU Munich, Munich, Germany; 2 AUVA Trauma Center Styria, Graz and Kalwang, Austria; 3 Department of Orthopaedic and Trauma Surgery Lucerne, Cantonal Hospital Lucerne, Luzern, Switzerland

**Keywords:** Handgrip strength, Dynamometer, Vitamin D, 25-OH vitamin D concentration, Orthogeriatric, Trauma patients

## Abstract

**Background and objectives:**

The treatment of elderly patients is an increasing challenge and the long-term sequelae often affect activities of daily living and quality of life in those patients. Handgrip strength (HGS) appears as a promising value to predict the outcome after trauma in elderly patients and to assess the overall muscle strength. Besides the possible role of psychological and hormonal factors, vitamin D may have a positive influence. Furthermore, some data suggest that Vitamin D is beneficial regarding muscle strength and possibly prevents further falls and injuries in orthogeriatric patients. The purpose of this study was to identify if Vitamin D is an influencing factor for HGSin elderly trauma patients.

**Materials and methods:**

94 elderly patients in a Level I Trauma Center aged 60 years or older were prospectively enrolled and HGS as well as serum 25-OH Vitamin D concentration (VDC) were measured. In addition, the standardized questionnaires Barthel Index (BI), Parker Mobility Score (PMS), Short Physical Performance Battery (SPPB), Strength, Assistance with walking, Rise from a chair, Climb stairs and Falls (SARC-F) and European Quality of Life 5 Dimensions 5 Levels Questionnaire (EQ-5D-5L), were used to record mental health status and demographic data.

**Results:**

HGS is mainly related to age and sex in elderly trauma patients. HGS was higher in men (mean_male_ = 27.31 kg (± 8.11), mean_female_ = 15.62 kg (± 5.63), *p* < 0.001 and decreased with age (β_age_ = − 0.58, *p* < 0.001). A significant negative correlation between HGS and VDC exists in the overall sample (β_VDC_ = − 0.27, p_VDC_ < 0.008), which still remains after adjusting for age (p_VDC_ < 0.004), but is not significant after adjustment for both main confounders, age and sex (p_VDC_ < 0.08). Furthermore, the HGS was lower in pateints who reported frequent falls, stumbling, dizziness or a late onset of menopause, and decreased if patients felt anxious or depressed during measurements (β_anxiety+depression_ = − 0.26, p_anxiety+depression_ < 0.01).

**Conclusions:**

These results do not support the hypothesis that Vitamin D has a positive influence on muscle strength measured by HGS. Nevertheless, this study could confirm the usefulness of HGS as a tool to detect the risk for frequent falls or stumbling. Furthermore, HGS seems to be associated with dizziness and age at onset of menopause. A significant decrease of HGS could also be shown in patients with anxiety and depression. This underlines the importance of interdisciplinary treatment of elderly trauma patients and needs to be taken into account for further studies, as especially the psychological motivation seems to have a significant influence and is sometimes not considered enough in elderly musculo-skeletal patients.

**Supplementary Information:**

The online version contains supplementary material available at 10.1186/s40001-023-01123-5.

## Introduction

The treatment of elderly trauma patients is an increasing socio-economic challenge and long-term sequelae often affect daily living and quality of life in those patients. Thereby, falls are a frequent reason for the presentation in a clinic. There are multiple reasons for elderly people to fall, such as visual and sensory deficits, muscle weakness and mobility impairment, diseases and side effects of medication [1]. In this regard, muscle strength is a very sensitive indicator of morbidity and mortality in older adults [2]. Loss of skeletal muscle mass, function and strength are the major characteristics of aging muscles [3]. Beginning at the age of 40, humans lose approximately 1–2% of muscle mass per year [4]. There are far less studies on the condition or progress of decreasing muscle strength compared to research on bone metabolism and osteoporosis in the elderly and accordingly scarce approaches for medical intervention. Improving muscle strength and counteracting sarcopenia is, therefore, an important goal of orthogeriatric treatment. Vitamin D could be a promising, widely available treatment with positive effects on sarcopenia.

Vitamin D is often presented as an agent with positive properties for the human organism and may have a positive influence on different organic systems as mental health, muscle mass or bone metabolism [6–8]. In a meta-analysis, Annweiler et al. found that vitamin D has a positive effect on gait speed in elderly patients and, thus, directly affects a vital sign positively [9].

In an animal experiment, Seelden et al. were able to show the positive effect of vitamin D and postulated that an increased vitamin D level could have positive effects in humans and may reduce frailty [5].

Considering the safe and reasonable way, vitamin D can be used as a supplementation, it has to be investigated if vitamin D, apart from its effects on bone metabolism, also affects muscle mass and strength and consequently can decrease the risk of falls in elderly trauma patients. This is even more interesting as vitamin D is taken daily by many elderly patients for the prophylaxis or treatment of osteoporosis.

To evaluate muscle strength in a trauma patient population, handgrip strength (HGS) is an established method [10]. HGS is recommended as a simple modality to assess muscle mass and function [11]. Additionally, the European and Asian working group on sarcopenia suggests the use of HGS to define sarcopenia in elderly people [14, 15]. Furthermore, HGS seems to be a promising tool to prognosticate the overall outcome in elderly patients and is already verified to predict the outcome after hip fracture [12, 13]. However, only few fall risk scores comprise mobility and balance tasks as well as a measurement of the dominant HGS [16].

There are already some studies covering the relationship between HGS and vitamin D concentration (VDC) but the results differ tremendously [8, 17]. While some of the studies claim a serious positive correlation [11, 17, 18], others identified no such relationship [19–23] or even found a high VDC associated with a lower HGS [23].

In contrast, Mendes et al. found an association between vitamin D deficiency and a lower gait speed as well as lower HGS in elderly patients [24]. Thus, the currently available data for a possible link between VDC and HGS are still concerning and no clear recommendation is possible. To the best of the authors` knowledge, there has been no study in elderly trauma patients that has examined the relationship of routine vitamin D supplementation because of osteoporosis and HGS as a “useful indicator for overall health” [2].

The major aim of this study was to investigate the relationship between HGS and vitamin D levels in elderly patients and moreover to identify potential other factors that may influence HGS in a collective of elderly trauma patients. The hypothesis is that higher vitamin D serum levels have a positive effect on HGS in elderly trauma patients and therefore, the supplementation of vitamin D is not only benefitable for the prevention of osteoporosis.

## Methods

### Study design and participants

Between November 2020 and April 2021, elderly trauma patients who were treated in a Level I Trauma Center with specialized orthogeriatric care were consecutively included in the investigation. Enrolled patients were treated mainly for proximal femur fractures (*n* = 43), followed by lower leg injuries (*n* = 18), mild traumatic brain injuries (GCS > 14, no intracerebral bleedings or traumatic brain injuries in the cranial CT scan) (*n* = 16) and fractures of the spine (*n* = 9). The study was approved by the local university ethics committee and registered under AZ 19-177. The study followed the Declaration of Helsinki. All patients were informed before inclusion in the study and written consent was obtained by every subject. Any Patient could consent to participate after a reflection period; patients under the care of a caregiver were not included.

Excluded were patients under 60 years of age, patients with a decreased cognitive status or injuries/diseases, which may result in a reduced HGS (post-stroke plegia, upper extremity fractures including forearm fractures and finger fractures, neuromuscular diseases as Parkinson, intracerebral injuries as bleedings or cranial fractures).

Besides the HGS and VDC demographical and medical data of the patients, their past and present physical activity, daily living skills and leisure-time activities were collected. Therefore, established surveys and scores such as Barthel Index [25], Parker Mobility Score [26], Short Physical Performance Battery [27], a screening test for persons with sarcopenia (SARC-F) [28] and the European Quality of life 5 Dimensions 5 Levels Questionnaire (EQ-5D-5L) [29] were used. Results were mostly self-reported by the patients, for example regarding the frequency of falls, stumbling, dizziness and age at onset of menopause.

Serum 25-OH-hydroxy-vitamin D level (VDC) was measured using the cobas^®^ 8000 e 801 (Roche Diagnostics, Germany) and expressed in nanogram per milliliter [ng/ml].

The HGS was assessed in kilogram [kg] using the DynEx^®^ Dynamometer (MD Systems Inc., USA), which is validated in several studies [13, 30]. After a standardized instruction, based on the key recommendations for HGS assessment of the American Society of Hand Therapists [31], patients were asked to take a seated position with shoulder adducted and in neutral rotation, elbow flexed at 90 degrees, wrist between 0 and 30 degrees of flexion and between 0 and 15 degrees of ulnar deviation. Following instruction through the staff, three attempts of maximal voluntary squeezing were performed on both hands. A sufficient rest period of minimally 15 s was provided between grip repetitions. The resulting HGS is the arithmetic mean of six measurements, three with the left and three with the right hand.

### Statistical analysis

The comparisons of variables in two populations (one of them with a special feature and the other population without it) were conducted with the two-tailed Student´s t-test whenever variables were normally distributed and the variances were equal. The required group size was determined using G*Power (Version 3.1; Heinrich-Heine University, Dusseldorf, Germany). For this purpose, a group size of at least 71 persons was calculated for an expected effect size *ρ* = 0.4 to achieve a possible statistical effect. Normal distribution was assessed using a Quantile–Quantile plot and equality of variances with the Levene test. When the Levene test delivered no equality of variances in these variables, the Welch test was performed. To assess for relationship between more than two variables or more than one numeric variable, multiple linear regression models were used. The level of significance was set at *p* < 0.05 and confidence interval (CI) at 95%. Results are shown as arithmetic mean (mean) ± standard deviation (SD), percentage (%) or standardized β coefficients (β). Graphs and statistical analysis were performed with R version 4.0.3 (2020-10-10) (R Core Team, 2020, R Foundation for Statistical Computing, Vienna, Austria).

## Results

After exclusion of all unsuitable patients, a total of 94 (62 females, 32 males) patients remained. In this study, the enrolled patients had a mean age of 78.5 (± 8.6) years. Detailed demographic data and patients` characteristics are displayed in Table 1. There was no significant influence on the outcomes by type of attendance (inpatients or outpatients), treatment method (operation or conservative), length of stay or possible ICU treatment. The means of patients` age, VDC and HGS are shown in Table 2.

**Table 1:** Demographic data and patients` characteristics

	Total	Female			Male		
		Total	Vitamin D substituted	Not vitamin D substituted	Total	Vitamin D substituted	Not vitamin D substituted
Total	94	62	34	28	32	10	22
Age							
60–69year	19	9	4	5	10	3	7
70–79year	23	13	8	5	10	6	4
80–89year	45	33	19	14	12	1	11
≥ 90year	7	7	3	4	0	0	0
Treatment method							
Operative	66	48	27	21	18	8	10
Conservative	28	14	7	7	14	2	12
Frequent falls							
Yes	28	19	11	8	9	5	4
No	66	43	23	20	23	5	18
Frequent stumbling							
Yes	21	15	8	7	6	2	4
No	73	47	26	21	26	8	18
Frequent dizziness							
Yes	27	15	9	6	12	4	8
No	67	47	25	22	20	6	14
Anxious/depressed							
Not	51	29	14	15	22	6	16
Slightly	29	21	11	10	8	3	5
Moderately	8	6	6	0	2	1	1
Severely	3	3	3	0	0	0	0
Extremely	1	1	0	1	0	0	0
Age at onset of menopause							
< 50 y	26	26	15	11	–	–	–
≥ 50 y	33	33	19	14	–	–	–

**Table 2:** Mean, standard deviation (SD) and 95% confidence interval (CI) of age, serum vitamin D concentration (VDC) and handgrip strength (HGS) in 94 included elderly trauma patients

	Sex	Vitamin D substitution	Mean	SD	CI	
Age [y]	Total		78.50	8.60	61.00	91.00
	Female		79.95	8.09	61.52	91.47
	Male		75.69	8.98	61.00	88.00
VDC [ng/ml]	Total		25.57	15.22	5.00	60.21
		Yes	35.57	13.45	12.82	61.62
		No	16.76	10.57	3.82	39.33
	Female		28.34	15.79	4.24	59.91
		Yes	37.15	12.72	13.76	60.88
		no	17.65	12.23	3.34	42.94
	Male		20.19	12.61	6.63	49.52
		Yes	30.21	15.19	13.89	59.58
		No	15.63	8.14	6.56	31.13
HGS [kg]	Total		19.60	8.59	6.21	39.00
		Yes	17.43	7.08	6.29	32.47
		No	21.52	9.38	6.76	42.95
	Female		15.62	5.63	6.11	27.11
	Male		27.31	8.11	15.87	43.91

### Influence of age and sex on HGS and VDC

A clear influence on the HGS was found for age and sex. Men has a significant higher HGS than women (mean_male_ = 27.31 kg, mean_female_ = 15.62 kg, *p* < 0.001). The higher the age of the patients, the lesser the HGS (β_age_ = − 0.58, *p* < 0.001). In addition, the VDC was significantly influenced by sex (mean_male_ = 20.19 ng/ml, mean_female_ = 28.34 ng/ml, *p* < 0.008), but not to the age of the patients.

### HGS and VDC

**Fig. 1:**
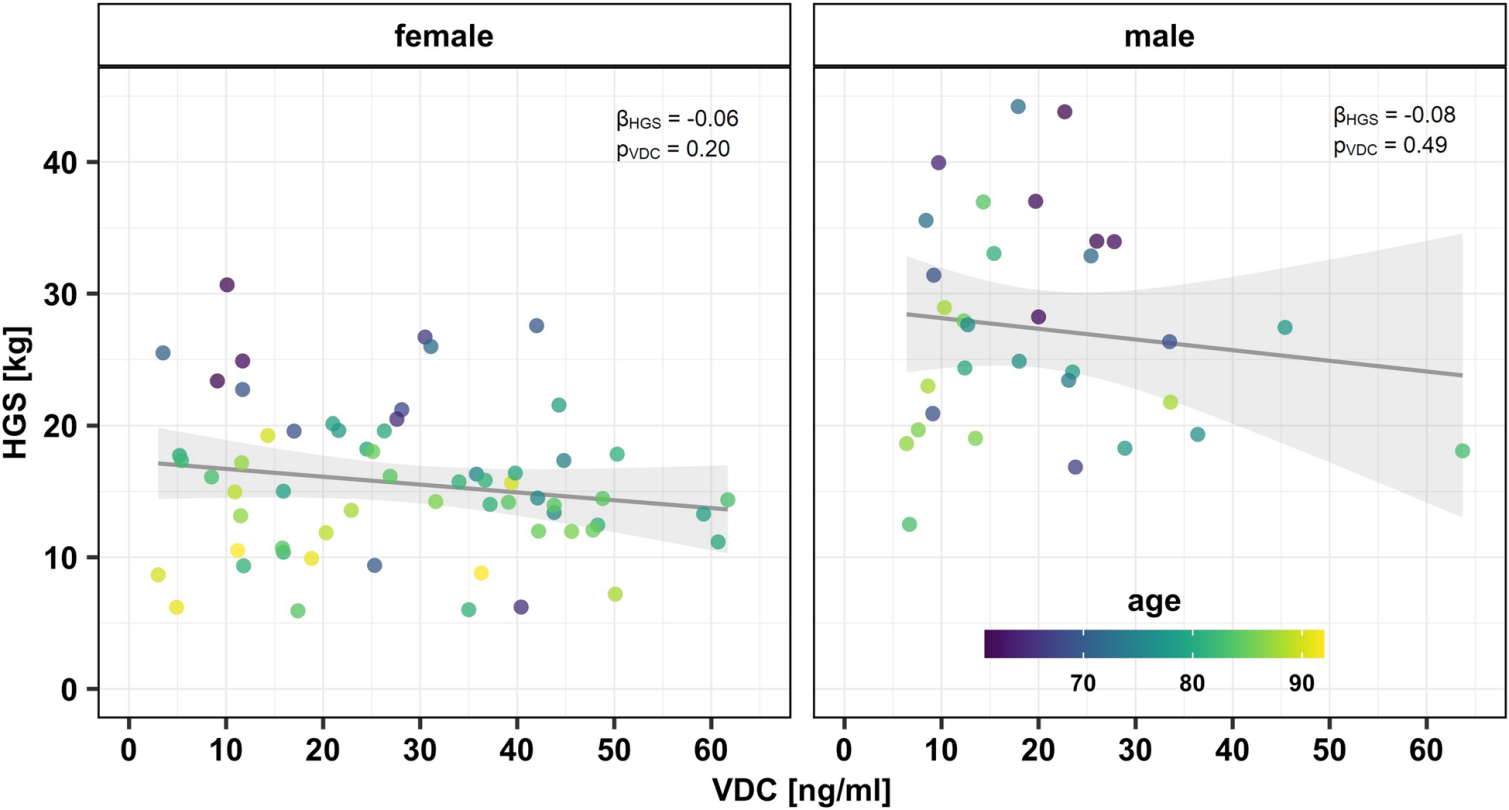
Handgrip strength (HGS) and serum 25-hydroxy-vitamin-D concentration (VDC) in 62 female and 32 male elderly traumatological patients; added fitted regression model lines with 95% confidence region (gray)

In total, a significant negative correlation was found between the HGS and the VDC (β_VDC_ = − 0.27, p_VDC_ = 0.008). The higher the VDC, the lower the HGS and vice versa (Fig. 1). This relationship becomes even more clear when adjusted for age (p_VDC_ = 0.004).

However, when sex is added as cofounder, the correlation between HGS and VDC is not significant (p_VDC_ = 0.08). Furthermore, there is no correlation if separately analyzed in sex-specific subgroups, neither in men (β_HGS_ = − 0.08, p_VDC_ = 0.49) nor in women (β_HGS_ = − 0.06, p_VDC_ = 0.20).

### Influence of vitamin D substitution

**Fig. 2:**
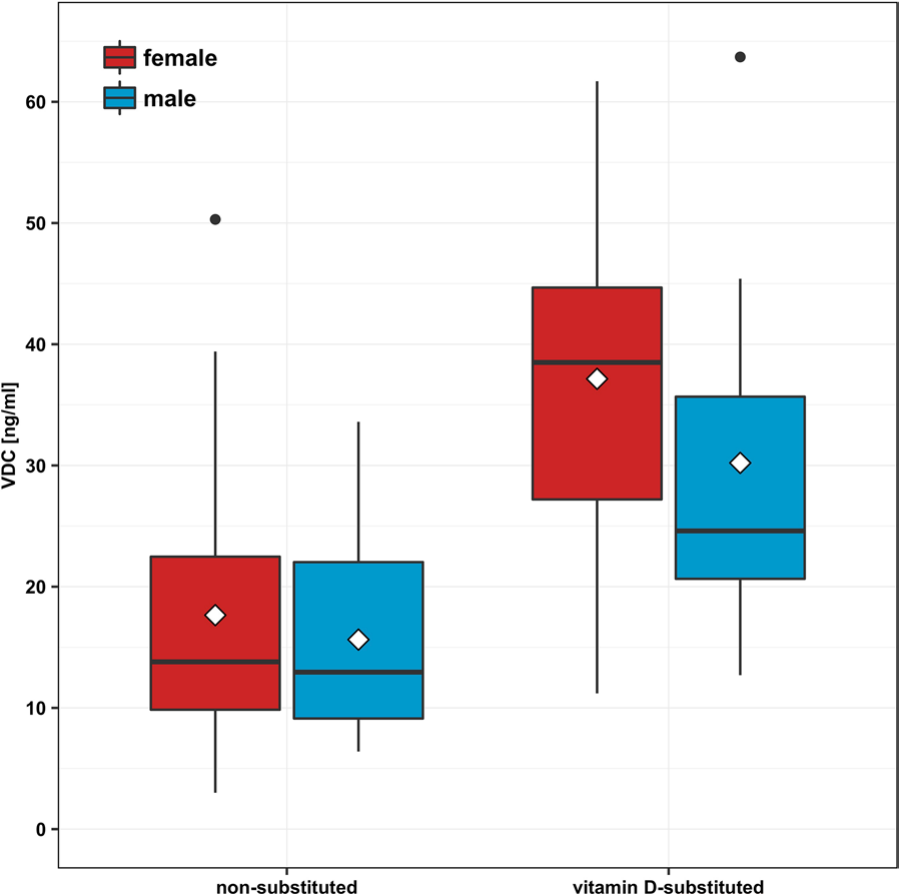
Serum 25-hydroxy-vitamin-D concentration (VDC) in vitamin D substituted and not substituted women and men in 94 elderly traumatological patients; box goes from 25 to 75th percentiles of the data, median = line, mean = diamond

Under vitamin D substitution were 54.8% of the included women, compared to 31.3% of the male patients. Substituted patients had a significant higher VDC than non-substituted patients (mean_female_vit_d_substituted_ = 37.15 ng/ml, mean_female_not_vit_d_substituted_ = 17.65 ng/ml, p_female_ < 0.001; mean_male_vit_d_substituted_ = 30.21 ng/ml, mean_male_not_vit_d_substituted_ = 15.63 ng/ml, p_male_ < 0.0013) (Fig. 2). The percentage increase in the VDC in substituted individuals compared to the VDC in non-substituted individuals was about the same in men, women and the overall sample (+ 93%, + 110%, + 112%).

As mentioned above, women in this study had a higher VDC. Considering that they also had a higher percentage of substituted individuals, there were no statistically significant differences in the VDC between men and women when analyzed in substitution- or non-substitution subgroups (mean_vit_d_substituted_males_ = 30.21 ng/ml, mean_vit_d_substituted_females_ = 37.15 ng/ml; mean_not_vit_d_substituted_males_ = 15.63 ng/ml, mean_not_vit_d_substituted_females_ = 17.65 ng/ml). Furthermore, a notable lower HGS was found in the substituted group compared to the non-substituted patients (mean_vit_d_substituted_ = 17.43 kg, mean_not_vit_d_substituted_ = 21.52 kg, *p* < 0.02). This finding persists when adjusted for age (β_substitution_yes/no_ = − 0.23, β_age_ = − 0.57, p_substitution_yes/no_ < 0.006), but is not significant anymore when sex is included (*p* < 0.08).

When taking a closer look at the non-substitution group, there is no significant correlation between HGS and VDC, even if adjusted to age and/or sex. Whereas the results in the substitution group are similar to the ones in the complete sample: In this group, HGS and VDC have a negative relationship (β_VDC_ = − 0.35, *p* = 0.02), which still remains significant if adjusted by age (β_VDC_ = -0.69, β_age_ = − 0.50, p_VDC_ < 0.04), but not if sex is added or the correlation is analyzed separately in women and men, respectively.

### Influence of functional variables on HGS

**Fig. 3:**
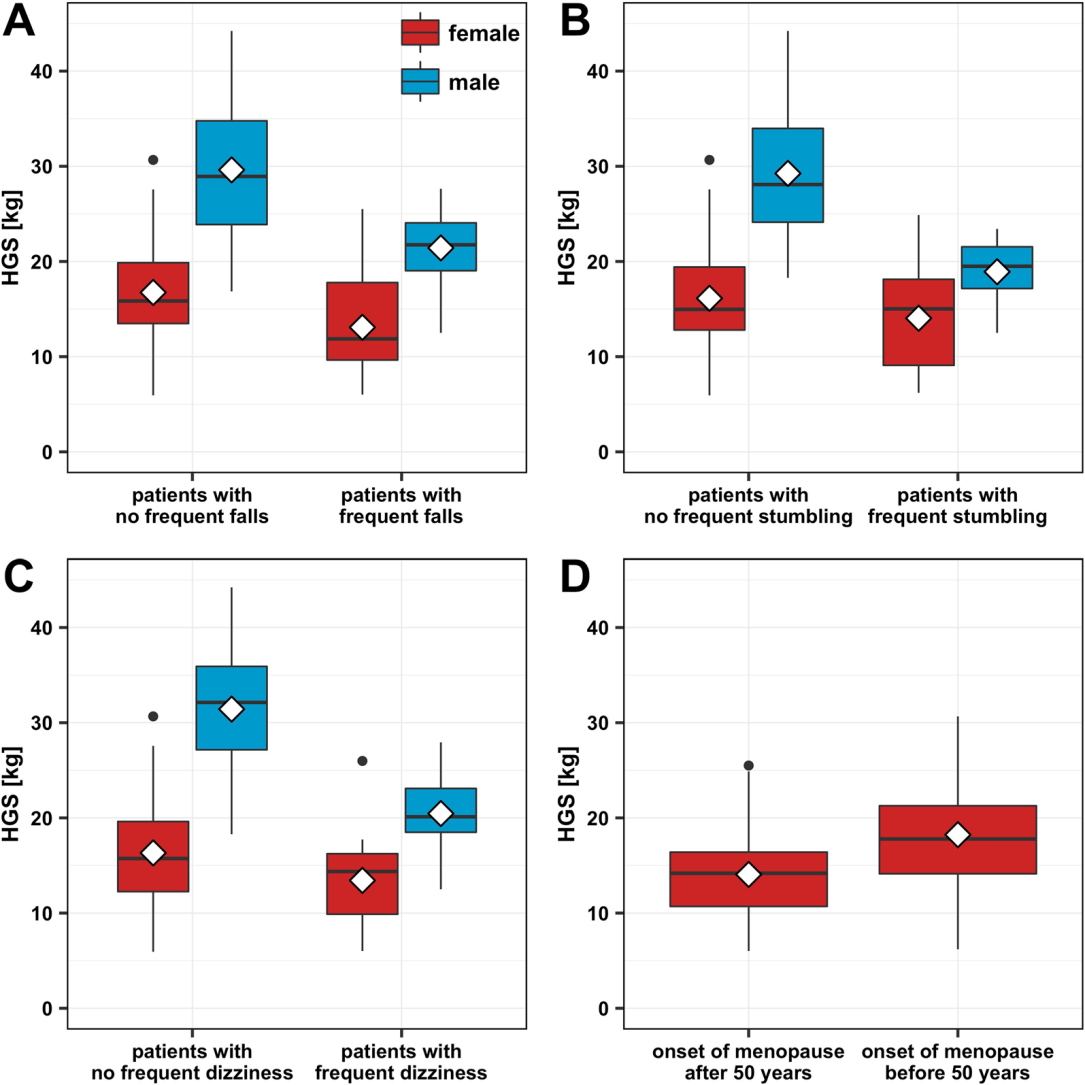
Handgrip strength (HGS) in patient cohorts with frequent falls (**A**), stumbling (**B**) or dizziness (**C**) in 94 elderly trauma patients and HGS in patients with early and late onset of menopause in 59 female elderly traumatological patients (**D**); box goes from 25 to 75th percentiles of the data, median = line, mean = diamond

This study found a statistically significant negative relationship between the HGS and a history of multiple falls (mean_falls_ = 15.76 kg (± 6.49), mean_no_falls_ = 21.23 kg (± 8.88), *p* < 0.004) (Fig. 3A) or frequent stumbling (mean_stumbling_ = 15.43 kg (± 5.61), mean_no_stumbling_ = 20.80 kg (± 8.94), *p* < 0.01) (Fig. 3B) in the last 12 months.

Likewise, the HGS was less if the patients reported frequent dizziness (mean_dizziness_ = 16.55 kg (± 5.89), mean_no_dizziness_ = 20.83 kg (± 9.21), *p* < 0.009) (Fig. 3C), especially among the male patients (mean_male_dizziness_ = 20.46 kg (± 3.98), mean_male_no_dizziness_ = 31.43 kg (± 7.12), *p* < 0.001).

Besides, a weak but nevertheless significant negative correlation between the HGS and the age at onset of menopause was found (β_menopause_ = − 0.26, p_menopause_ < 0.04). If separated into groups depending on the reported age at onset of menopause (mean_age_at_menopause_ = 48.9 y (± 6.0)), patients who got an earlier menopause (before 50 years of age), had a notable higher HGS (mean_menopause_before_50_ = 18.23 kg (± 5.73), mean_menopause_≥50_ = 14.06 kg (± 4.78), *p* < 0.004) (Fig. 3D).

**Fig. 4:**
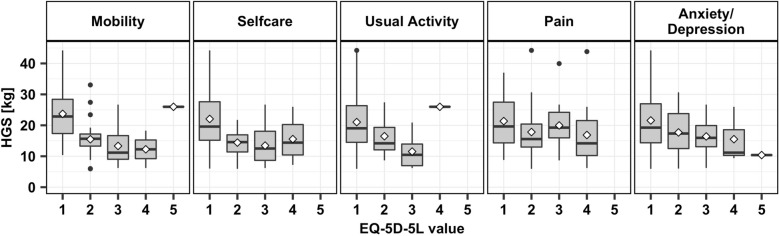
Handgrip strength (HGS) in the different EQ-5D5L values for 92 elderly trauma patients; box goes from 25 to 75th percentiles of the data, median = line, mean = diamond

The more anxious and depressed the patients felt in the moment of measurement, recorded by the EQ-5D-5L, the lower their HGS was (β_anxiety+depression_ = − 0.26, p_anxiety+depression_ < 0.01) (Fig. 4). A clear tendency can be seen not only for the depression item, but also for all others. The poorer values in EQ-5D-5L are associated with reduced HGS. This effect becomes even more obvious if adjusted by age and sex (β_anxiety+depression_ = − 0.18, β_age_ = − 0.48, β_sex_ = 0.50, p_anxiety+depression_ < 0.008).

Analysis of other additional information collected on subjects' demographic and medical data, their past and present physical activity, daily living skills, and leisure-time activities, measured with Barthel Index, Parker Mobility Score, Short Physical Performance Battery, SARC-F did not reveal any new significant associations (See also: Additional file 1: Figs. S1 and S2).

## Discussion

The main purpose of this study was to investigate the relationship of HGS and vitamin D levels in elderly trauma patients. This is of particular interest due to the potential muscle anabolic properties of vitamin D and, thus, a possible starting point to prevent sarcopenia and frailty. However based on the study population, no positive influence of vitamin D level on muscle strength could be found. Nevertheless, a significant influence of subjective patient data on HGS could be found. In particular, the psychological condition seems to have a significant influence on muscle function in elderly trauma patients as well as the frequency of falls and stumbling, dizziness and the age at onset of menopause.

There is weak evidence in the literature for a muscelanabolic effect of vitamin D. Thus, Chiang was able to demonstrate muscle anabolic effects in particular for vitamin D3 in a metanalysis [8]. Another meta-analysis by Halfon et al. concluded that elderly patients in particular benefit from vitamin D, and muscle strength and gait benefit from regular vitamin D administration [17]. However, in most studies, results were only slightly significant or solely notable in especially generated subgroups, e.g., specific levels of VDC or special age groups [34]. The authors could not confirm any of these findings in their study population. In contrast, substituted individuals in this study had a notable lower HGS and the higher the vitamin D level, the lower the HGS. These effects may be attributed by the fact that mainly females and older patients, who usually have a lower average HGS, got vitamin D substitution and consequently had a higher VDC. Also, the potentially lower physical activity of patients receiving vitamin D substitution could be a possible influencing factor for the lower HGS. Patients often receive vitamin D routinely only after trauma or fracture. This trauma could have reduced activity and therefore muscle weakness and a lower HGS. An age- and sex-independent analysis of the results also showed no association between HGS and VDC in our patient population. In this way, the present research supports the findings of other studies, which claim no significant correlation between VDC and HGS, neither analyzed as a comparison of substituted and non-substituted individuals [20, 21] nor referred to the individual serum level [22, 23]. Additionally, a systematic review on the association of vitamin D and HGS observed no significant improvement of HGS after administration of vitamin D supplements [19].

Even if vitamin D may have some effects on muscles on cellular level [17], HGS and its measurement are influenced by a quantity of parameters and VDC is a rapidly changing and easily influenced factor [8]. The fact that only small differences in the study settings can cause completely opposite results [19] may be an important hint that there is no serious significant relationship between HGS and VDC. Just by minor variations in variables regarding the selected participants (e.g., basis VDC, age group or special characteristics of chosen individuals), and vitamin D substitution (e.g., dose, frequency) major changes in the outcomes of the studies could be observed.

HGS is also a commonly used method to detect frailty or an increased risk for falls [12, 17, 35]. The present study could confirm these results and approve the benefit of HGS as a reliable screening tool. From our point of view, it is also easy and reliable to use in everyday clinical practice. In contrary to many other studies [32], HGS in this study was measured on both hands and each side multiple times, which may give a better comparable average. HGS measurement is said to be a very convenient method available in clinical practice and the data is appreciated as highly reliable and valid [33]. Furthermore, there seems to be an interaction of dizziness and HGS. Whether frequent dizziness reduces patients` mobility and as a result of less activity decreases the HGS, a lack of mobility is the cause of dizziness or if there is another totally different etiology, cannot be answered with this study setting. Considering that dizziness is a common symptom, which up to 20% of elderly patients experience as severe enough to affect their daily activities, more specific research should be done on this issue [36].

Another important aspect is the influence of depression or anxiety while measuring HGS on the outcome. This study found a notable difference in HGS depending on the level of depression and anxiety. The more depressed or anxious patients were, the less was their HGS, which may be due to declined motivation. This association between HGS and depression was also demonstrated in a large registry study by Brooks et. al. However, the possible biochemical background is still completely unclear. A possible link between sarcopenia and depression could be impaired mitochondrial function or a chronic inflammatory response [37]. Therefore, the authors strongly recommend taking the psychological aspect into account when performing research that is based on collaboration of participants.

Still not sufficiently investigated is the relationship of HGS and age at onset of menopause. Already existing studies mainly analyze muscle mass and strength in postmenopausal women [38, 39]. These studies more or less agree that (post-)menopause is associated with lower HGS [38]. They also claim (post-)menopause to be an additional factor on top of age, due to the fact that HGS—or muscle mass and strength in general—decrease in both, women and men, but notable greater and more accelerated in women [40]. This may be attributed by various effects depending on changes in the hormone status of postmenopausal women, especially estrogen decline. Studies which found a positive effect of hormone replacement therapy on muscle force support this thesis. [38]. Considering the importance of postmenopausal effects for females, the consequences of women’s age at onset of menopause should be further investigated.

## Limitations

Although the authors collected and analyzed much more information apart from HGS and VDC, some details, especially data referring to patients` recent daily activity may be influenced due to COVID-19 pandemic. Furthermore, the handle of the DynEx^®^ Dynamometer was not adjustable and patients—especially women with smaller hands—were found to be struggling to get a good grip position. This was complained about by some patients. Anyway, the main limitation of this study is the small number of patients, especially men. The smaller the sample size the more difficult it is to detect possibly existing weak relationships between VDC and HGS. Further longitudinal studies with a larger sample size are needed to guarantee the care and attention the topic deserves.

## Conclusions

In this study, no evidence was found that there is a positive correlation between HGS and VDC in elderly trauma patients. However age, sex, mental health and onset of menopause are strongly associated with HGS. Therefore in our collective, the muscle anabolic effect does not play a role in routine daily vitamin D supplementation. Further studies on this topic are needed, including larger samples and preferably longitudinal, randomized, site-controlled trials, to clarify whether a much higher dose has a muscle anabolic effect and, thus, may reduce the risk of falls.

## Supplementary Information

**Additional file 1.** **Figure S1.** Correlation matrix of handgrip strength (HGS) and other collected data. Red= negative correlation, green = positive correlation.

**Additional file 2.** **Figure S2.** Relationship of handgrip strength (HGS) and score of Barthel Index (range from 0 = worst to 100 = best) (A), Parker Mobility Score (range from 0 = worst to 9 = best) (B), Short Physical Performance Battery (range from 0 = worst to 12 = best) (C) and SARC-F (range from 0 = best to 10 = worst) (D). Box goes from 25th to 75th percentile of the data, median = line, mean = diamond.

## Data Availability

The data presented in this study are available on request from the corresponding author. The data are not publicly available due to privacy and data protection regulations.
