# Supplementary figures and images for: The influence of vitamin D on handgrip strength in elderly trauma patients
Source: Eur J Med Res. 2023 May 13;28:170. doi: 10.1186/s40001-023-01123-5 (PMC10181921; doi:10.1186/s40001-023-01123-5)

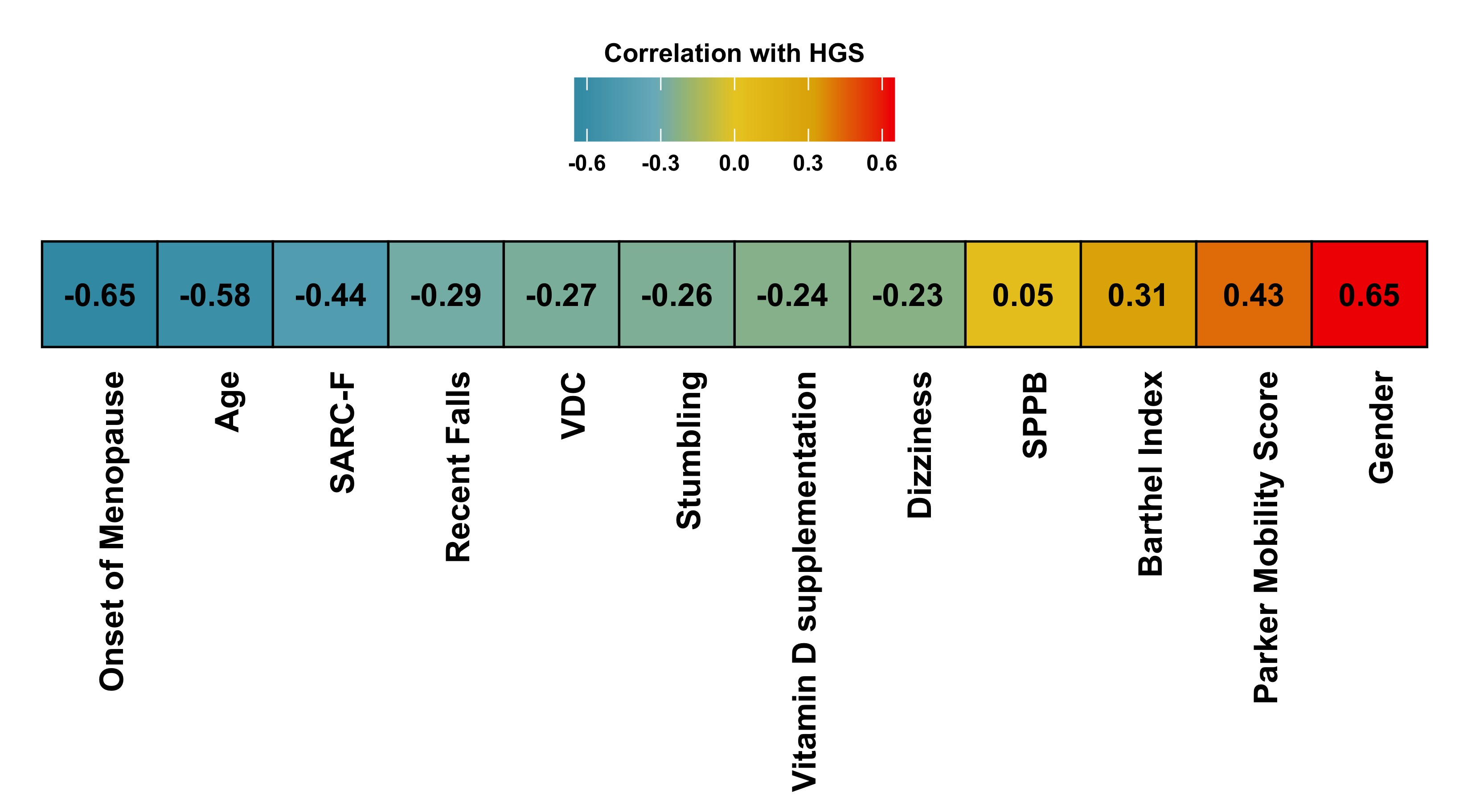

Supplement: Supplementary file 1 — Additional file 1. Figure S1. Correlation matrix of handgrip strength (HGS) and other collected data. Red= negative correlation, green = positive correlation. [file 40001_2023_1123_MOESM1_ESM.jpg]

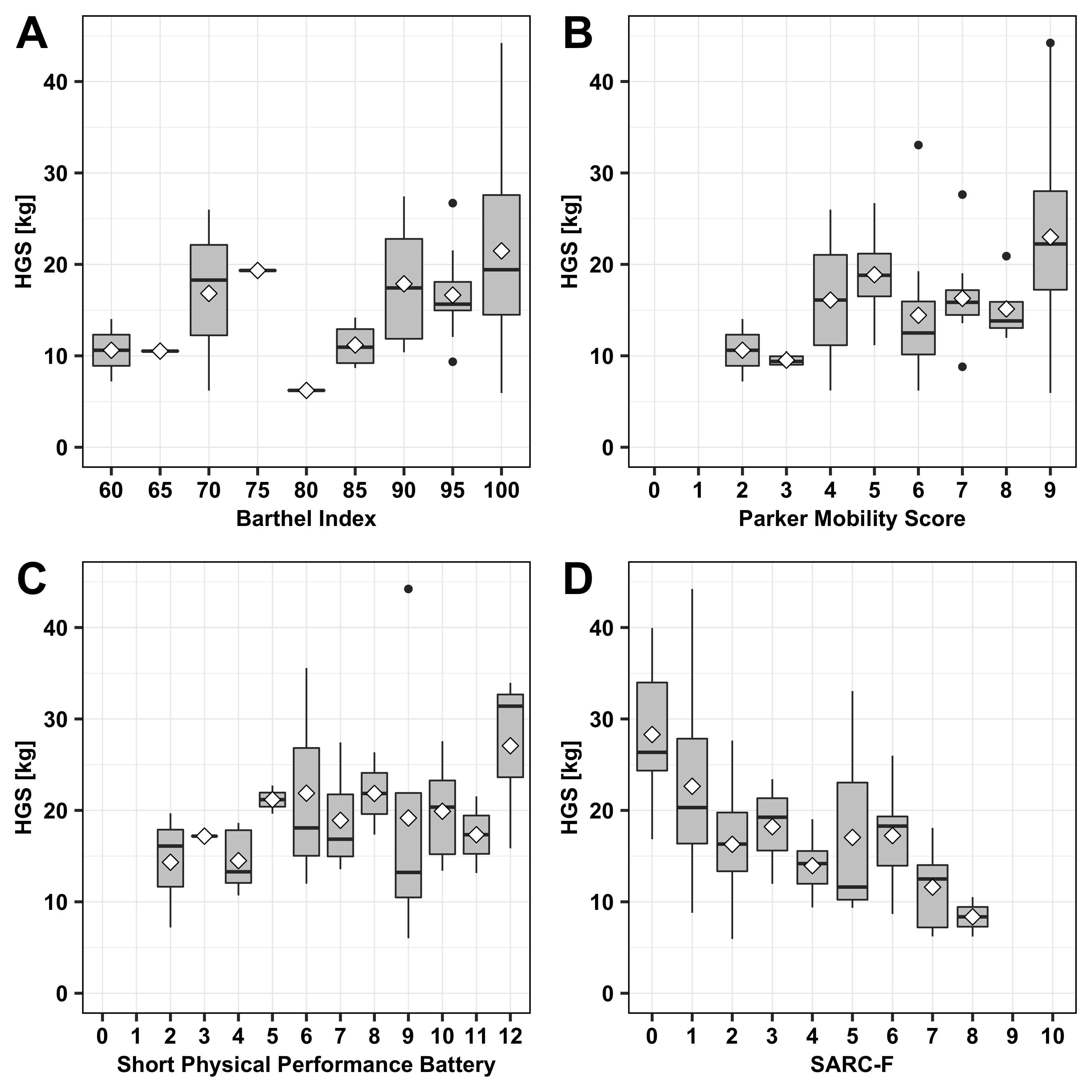

Supplement: Supplementary file 2 — Additional file 2. Figure S2. Relationship of handgrip strength (HGS) and score of Barthel Index (range from 0 = worst to 100 = best) (A), Parker Mobility Score (range from 0 = worst to 9 = best) (B), Short Physical Performance Battery (range from 0 = worst to 12 = best) (C) and SARC-F (range from 0 = best to 10 = worst) (D). Box goes from 25th to 75th percentile of the data, median = line, mean = diamond. [file 40001_2023_1123_MOESM2_ESM.jpg]
